# Remote psychoeducation for eating disorders: An exploratory study during lockdown

**DOI:** 10.1192/j.eurpsy.2021.1858

**Published:** 2021-08-13

**Authors:** L. Cruchet, E. Scanferla, A. Laszcz, P. Gorwood, L. Romo

**Affiliations:** 1 Ea 4430 Clipsyd, Department Of Psychology, Université Paris Nanterre, Nanterre, France; 2 Ed 450, Université de Paris, Paris, France; 3 Ea 4403, Clipsyd, Université Paris Nanterre, France, France; 4 Cmme, GHU Paris psychiatrie neurosciences, Paris, France; 5 Institute Of Psychiatry And Neuroscience Of Paris, INSERM, Paris, France

**Keywords:** COVID-19, psychoeducational intervention, remote intervention, eating disorders

## Abstract

**Introduction:**

With increasing prevalence, eating disorders (EDs) constitute a public health problem. Access to treatment is limited and often delayed for the majority of patients. Such obstacles might be mitigated via the development of virtual treatments.

**Objectives:**

Conducted during COVID-19 lockdown, this pilot study aimed to explore the feasibility and preliminary clinical outcomes associated with treatment of EDs by means of a remote psychoeducational (PE) programme.

**Methods:**

Eleven patients who fulfilled DSM‐5 criteria for anorexia nervosa, bulimia nervosa, or binge eating disorder completed assessments, including ED symptoms, anxiety and depression, as well as motivation to change measures at the beginning and end of the time-limited (4 weeks) specialized treatment. It consisted in receiving 4 PE documents by email (1 per week), which was completed by a 15-20 minutes phone call with each participant (1 per week).

**Results:**

Data showed significant improvements of several self‐reported eating disorder symptoms, including body dissatisfaction and intensity of bulimic episodes.

**Conclusions:**

Our findings suggest that a time-limited remote PE intervention produces clinically meaningful changes in ED symptoms. Thus, it might be worth developing such interventions in a clinical context, especially when performed prior to higher level of care. Further research is required to evaluate optimised interventions using a more diverse sample from a plurality of treatment facilities and context of care, as well as research in a non-pandemic setting which may have impacted these exploratory study results.

**Disclosure:**

No significant relationships.

